# The Impact of Medicaid Expansion on Patients with Cancer in the United States: A Review

**DOI:** 10.3390/curroncol30070469

**Published:** 2023-07-02

**Authors:** Alexandra Hotca, Julie R. Bloom, Juliana Runnels, Lucas Resende Salgado, Daniel R. Cherry, Kristin Hsieh, Kunal K. Sindhu

**Affiliations:** Department of Radiation Oncology, Icahn School of Medicine at Mount Sinai, New York, NY 10029, USA; alexandra.hotca-cho@mountsinai.org (A.H.); julie.bloom2@mountsinai.org (J.R.B.); juliana.runnels@mountsinai.org (J.R.); lucas.resendesalgado@mountsinai.org (L.R.S.); daniel.cherry@mountsinai.org (D.R.C.); kristin.hsieh@mountsinai.org (K.H.)

**Keywords:** Medicaid expansion, cancer patients

## Abstract

Since 2014, American states have had the option to expand their Medicaid programs as part of the Affordable Care Act (ACA), which was signed into law by former President Barack H. Obama in 2010. Emerging research has found that Medicaid expansion has had a significant impact on patients with cancer, who often face significant financial barriers to receiving the care they need. In this review, we aim to provide a comprehensive examination of the research conducted thus far on the impact of Medicaid expansion on patients with cancer. We begin with a discussion of the history of Medicaid expansion and the key features of the ACA that facilitated it. We then review the literature, analyzing studies that have investigated the impact of Medicaid expansion on cancer patients in terms of access to care, quality of care, and health outcomes. Our findings suggest that Medicaid expansion has had a positive impact on patients with cancer in a number of ways. Patients in expansion states are more likely to receive timely cancer screening and diagnoses, and are more likely to receive appropriate cancer-directed treatment. Additionally, Medicaid expansion has been associated with improvements in cancer-related health outcomes, including improved survival rates. However, limitations and gaps in the current research on the impact of Medicaid expansion on patients with cancer exist, including a lack of long-term data on health outcomes. Additionally, further research is needed to better understand the mechanisms through which Medicaid expansion impacts cancer care.

## 1. Introduction

The passage of The Patient Protection and Affordable Care Act (ACA) in 2010 heralded a substantial shift in U.S. healthcare policy. The ACA’s primary aim was to expand access to affordable and comprehensive healthcare coverage to millions of uninsured Americans, a goal partially realized through the expansion of state Medicaid programs. This reform has resulted in a wealth of research investigating its implications across various health domains, with an increasing body of evidence detailing its impact on cancer care. Cancer, an affliction that presents considerable financial burdens to patients and their families, serves as a critical case study for assessing the effects of Medicaid expansion.

This review aims to review the existing research surrounding the effects of Medicaid expansion on patients with cancer in the United States. To contextualize these findings, we first outline the historical trajectory of Medicaid expansion and delineate the key provisions of the ACA that have enabled this policy shift. We then explore the literature examining the consequences of Medicaid expansion on patients with cancer. We focus, in particular, on its effects on the accessibility of care, the quality of care delivered, and overall health outcomes. Crucially, our review also addresses the limitations and gaps in the current research landscape, including the absence of long-term health outcomes data and the need for a more thorough understanding of the mechanisms by which Medicaid expansion exerts its impact on cancer care. By highlighting these limitations, we aim to foster a comprehensive understanding of the policy’s implications and guide future research directions in this critical area of health policy.

## 2. Medicaid Expansion: A Brief History

In the wake of the Great Depression and World War II in the mid-twentieth century, federal funds were, for the first time, allocated to states to promote the health of certain populations, including seniors, children, and caretakers [1]. In 1965, Medicaid and Medicare were enacted under Title XVIII and Title XIX of the Social Security Act. Over the years, additional groups of individuals have been made eligible for Medicaid coverage, including members of low-income families, individuals with disabilities and in need of long-term care, pregnant women, and the unemployed, among others. Additionally, the federal government has attempted to standardize the basic minimum services required for coverage over the years, which have come to include periodic screening examinations in children, preventive care services, and emergency medical treatment. At the same time, however, states have retained the ability to opt in and tailor their Medicaid programs to the individual needs of their citizens.

However, despite these national efforts to expand healthcare coverage, access to affordable and high-quality care in the United States varied widely prior to the enactment of the ACA. Health insurance coverage varied widely across a number of demographic factors, including, but not limited to, ethnicity, geography, race, sex, and socioeconomic status. While the uninsured rate had fallen dramatically from 1960 to 2000, from greater than 30% to approximately 14% [2], 46.3 million individuals living in the U.S. remained uninsured in 2009 [3]. Thus, a key goal of any new healthcare law would be the expansion of access to health insurance coverage.

## 3. Key Features of the ACA That Enabled Medicaid Expansion

In 2010, the ACA was signed into law with the aims of expanding access to health insurance, increasing consumer protection, improving quality and system performance, emphasizing prevention and wellness, expanding the healthcare workforce, and mitigating rising healthcare costs [4]. The ACA mandated that U.S. citizens and legal residents possess health insurance or face a tax penalty. In part to facilitate this, the bill expanded Medicaid coverage to adults under the age of 65 with incomes of up to 138% of the federal poverty level, thereby allowing for the largest gain in coverage for previously uninsured civilians [5]. Prior to the enactment of the ACA, specific federal programs provided coverage to the children of parents with limited incomes [6]. In expanding Medicaid eligibility, the ACA significantly expanded health insurance coverage to adults and led to an anticipated increase in the coverage of 17 million non-elderly adults. 

## 4. Current Status of Medicaid Expansion

The ACA mandated Medicaid expansion in every state and the District of Columbia (DC). However, a U.S. Supreme Court ruling in 2012, i.e., the National Federation of Independent Business v. Sebelius, made state participation optional [7]. Thus, only slightly more than half of the states and Washington, DC expanded Medicaid in 2014, with a net estimated total of 16.9 million individuals gaining insurance coverage in the process [8]. The ACA financially incentivized states to expand Medicaid. In fact, the federal government covered 100% of the health care costs of the expansion’s enrollees from 2014 to 2016, with this share decreasing only slightly beginning in 2017 before settling at 90% in 2020 [9,10]. By 2021, approximately three-quarters of the states chose to expand their Medicaid programs [11]. 

In order to encourage the remaining twelve states to expand their Medicaid programs, the American Rescue Plan Act (ARPA) of 2021 provided an additional financial incentive [12]. Specifically, the ARPA made states that were newly expanding their Medicaid programs eligible to receive an additional 5% increase, for two years, in their traditional Federal Medical Assistance Percentage (FMAP) [13,14]. The traditional FMAP, which is the percentage of each state’s total Medicaid expenditures that is reimbursed by the federal government, applies to most services for individuals in non-expansion groups, including children, most adults, and seniors. The two-year, 5% increase in the traditional FMAP remains available for any state that chooses to implement Medicaid expansion in the future and remains a significant inducement. In fact, a 2021 KFF analysis found that the two-year increase in the traditional FMAP would offset the cost of at least two years of Medicaid expansion in every state that had not yet expanded Medicaid [13]. 

**Figure 1:**
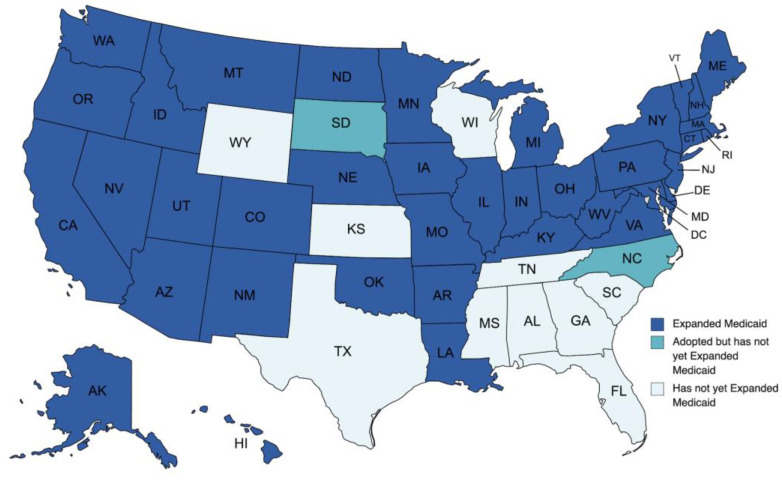
Current Status of Medicaid expansion among the US states as of April 2023: Data Derived from the Kaiser Family Foundation [11].

As of April 2023, 40 states and Washington, DC have formally adopted expansions of their Medicaid programs. Two of these states—including North Carolina, whose implementation is contingent on the biennial budget, and South Dakota, whose implementation is planned for July 2023—have not yet implemented their expansions [11]. The ten states that have not yet adopted the expansion of their Medicaid programs are Alabama, Florida, Georgia, Kansas, Mississippi, South Carolina, Tennessee, Texas, Wisconsin, and Wyoming, as illustrated in Figure 1. The path to Medicaid expansion in these states remains unclear. However, possible actions can still be made by the states’ executive branch, legislative branch, and/or voters to fulfill the ACA’s aim of universal Medicaid expansion.

## 5. Impact of Medicaid Expansion on Health and Hospitals

While Medicaid expansion initially faced some criticism in regard to its costs and effectiveness, studies over the past decade have shown that it has been broadly beneficial in many different areas, including improvements in access to care, quality of care, and even hospital finances [15]. Post-ACA studies of low-income adults in Medicaid expansion states showed significant increases in outpatient healthcare utilization, preventive care, health care quality, and self-reported health, as well as reductions in emergency department use compared to individuals in states that elected not to expand Medicaid [16]. Interestingly, however, another study from Oregon found that Medicaid coverage resulted in an increased overall emergency department use, relative to an average of 1.02 visits per person, by 0.41 visits per person or 40% [17].

A systematic review examining the effects of Medicaid expansion on access to care showed that expansion was associated with increased insurance coverage for all eligible individuals regardless of age, ethnicity/race, income, or marital status. Coverage gains, in fact, appear most pronounced in adults without a college degree. These gains, in turn, have led to increased primary care, mental health, and preventative visits [15].

Medicaid expansion has also had an impact on the quality of health care delivered. Studies indicate that Medicaid expansion is associated with improvements in glucose monitoring, the control of hypertension, prostate cancer screening, PAP smear screening, and—remarkably—post-operative complications [16,18,19,20]. Studies focusing on other health care metrics, such as self-reported psychological stress and mental health, also show improvements in the states that expanded their Medicaid programs [21,22]. Additionally, and perhaps most impressively, a large analysis of the literature exploring the effects of Medicaid expansion did not reveal any studies in which patients’ self-reported health status or outcomes were worse post-expansion [15].

Hospital finances have also been positively affected by Medicaid expansion. Most notably, uncompensated care has decreased, and Medicare revenues have risen according to some studies [23]. However, this may have come with the caveat that the costs of bad debt at non-profit hospitals may have increased [24]. Moreover, Medicaid expenditures did increase as a result of the expansion [25].

The overall effects of Medicaid expansion in the states that chose to implement it appear to be positive, with an increase in access to care across the board. Additionally, the data seem to indicate that increased availability did not lead to worsened healthcare quality. In fact, improvements in several health-related metrics have been noted after expansion.

## 6. Impact of Medicaid Expansion on Patients with Cancer

### 6.1. Improved Access to Care

The first two primary goals of the ACA—more affordable insurance for those who can pay, and expansion of Medicaid for those who cannot—represent two approaches in achieving the same objective: increasing the number of Americans with access to healthcare. With respect to this broad objective, the ACA was successful. In a study drawing on the National Health Interview Survey, the largest longitudinal health survey dataset in the United States, Chen et al. assessed changes in the probability of being insured from 2011–2014 alongside a variety of demographic and medical variables. When stratified by race/ethnicity, age, income, education, geographic region, and six chronic health conditions, all groups had a significant decrease in their probability of being uninsured from 2011–2014 [26].

Though improvements in access were broad, they were not felt uniformly. When compared to non-Latino Whites, improvements in uninsured rates were greater in African Americans (coef. = −0.04, *p* < 0.001) and Latinos (coef. = −0.03, *p* < 0.001). In the ensuing years, stratification by the decisions of states on whether to expand Medicaid showed that these decisions were an important tool through which to begin to decrease the longstanding disparities in uninsured rates. This finding can be observed in patients with cancer specifically. Among those with newly diagnosed cancer, the percentage of uninsured patients improved most in states that expanded Medicaid access (2.6% vs. 1.3%, *p* < 0.001). Furthermore, the rates of improvement in the percentage of uninsured patients were greater in Black (4.1% vs. 1.1%, *p* < 0.001), Hispanic (4.0% vs. 1.4%, *p* < 0.001), and rural patients (4.3% vs. 0.7%, *p* < 0.001) in Medicaid expansion states than their counterparts in non-Medicaid expansion states. Collectively, these changes had the impact of decreasing disparities in access to care in patients with cancer in states that opted to expand Medicaid, while such disparities were left relatively unchanged in the states that opted out of doing so [27].

The ACA’s implications for cancer care extend well beyond Medicaid expansion, however. A hallmark of the ACA felt acutely by cancer survivors is protection from insurers using a pre-existing cancer diagnosis to deny coverage, to instate a coverage denial period, or charge more for routine coverage. The ACA also required select private insurers to grant access to approved cancer clinical trials to their buyers, a benefit that had previously only been guaranteed to Medicare and Medicaid patients. Kehl et al. note that before the passage of the ACA, states had instituted a patchwork of policies that resulted in heterogeneous access to trials for privately insured patients, causing a small but statistically significant difference in approval rates between the privately and publicly insured (93% vs. 97%, respectively, *p* < 0.001) [28]. A criticism of the ACA is that it also set into law certain provisions that could permanently limit the broad realization of access to clinical trials among patients with cancer. Insurance plans that pre-date the ACA have, in some instances, been “grandfathered” into being able to evade provisions that require coverage for routine cancer care associated with clinical trial participation. Patients’ enrollment in these plans remains one of the most common reasons for their denial to participate in clinical trials [29].

### 6.2. Enhanced Quality of Care

High-quality cancer treatment is timely, appropriate, safe, and comprehensive. Medicaid expansion has been associated with earlier diagnoses, fewer treatment delays, and more treatment options being available for patients with cancer. An association between Medicaid expansion and an increase in early-stage diagnoses has been reported in patients with breast cancer [30], squamous cell carcinoma of the head and neck [31], gynecologic cancer [32], intrahepatic cholangiocarcinoma [33], hepatocellular carcinoma [34], gastric cancer [35], kidney cancer [36], pancreatic cancer [37], and melanoma [38]. Medicaid expansion has also been associated with an improved time in the initiation of treatment for Black and Hispanic patients with early-stage breast cancer who require adjuvant chemotherapy [39], and for patients with non-oropharyngeal HNSCC [40] and gynecologic cancers [32]. In an analysis of the receipt of timely systemic treatment for patients with advanced or metastatic cancers, Medicaid expansion was associated with reduced Black–White racial disparities [41].

Several studies suggest that there are better treatment options available to patients in Medicaid expansion states. In a study of patients with colorectal cancer, for example, Medicaid expansion was associated with an increase in local excisions (OR: 1.39, 95% CI: 1.13–1.69) and decreased rates of emergent surgery (OR: 0.85, 95% CI: 0.75–0.97) [42]. Medicaid expansion was also associated with an increase in the receipt of surgical treatment for patients with intrahepatic cholangiocarcinoma (OR: 1.19, 95% CI 1.03–1.38) (*p* < 0.01) [33]. Additionally, among the patients living in Medicaid expansion states, those with metastatic pancreatic cancer were more likely to receive palliative therapies [43], and patients with metastatic melanoma were more likely to receive immunotherapy [44]. Additionally, compared to those living in non-expansion states, a significantly higher percentage of patients in expansion states underwent post-mastectomy breast reconstruction (28.7% vs. 38.5%, *p* < 0.001) [45]. It has also been shown that cancer survivors in expansion states are more likely to have access to primary-care-based smoking cessation assistance for tobacco use disorder [46].

### 6.3. Positive Health Outcomes

Given the data supporting improved access to better quality care for patients with cancer since the enactment of Medicaid expansion, a careful analysis of treatment outcomes is essential to properly assess the impact of this policy change. In a recent study, survival trends were compared for patients with all cancer types, both individually and combined, residing in states that expanded Medicaid access and in states that did not. Patients in expansion states who were diagnosed with 15 of the 19 cancer types that are examined in this study had a net improvement in prognosis relative to their counterparts in nonexpansion states. This improvement was statistically significant in multivariable adjusted models for lung and bronchus cancers, non-Hodgkin lymphoma, liver and bile duct cancers, and pancreatic cancers [47]. Similarly, the research conducted by Han et al., demonstrated a significant correlation between the expansion of Medicaid and an enhanced increase in the two-year overall survival in newly diagnosed cancer patients [48]. This increase was especially notable among non-Hispanic Black individuals and patients who were living below 5% of the poverty line.

This survival benefit has been studied across many types of cancers. Medicaid expansion was associated with improved post-operative survival in patients with endometrial and ovarian cancer [49]. In patients with newly diagnosed stage IV breast cancer, Medicaid expansion was linked to enhanced survival rates and a reduction in the 2-year mortality gap. Though a disparity in survival rates between racial and ethnic minority groups and White patients was noted in the pre-expansion period, this was no longer evident in the post expansion period [50]. Among patients with colorectal cancers, patients in expansion states have been shown to have improved 90-day mortality rates (OR: 0.75, 95% CI: 0.59–0.97) and improved 5-year overall survival (hazard ratio (HR): 0.88, 95% CI: 0.83–0.94) [42]. Medicaid expansion was also associated with improvements in 1-year survival among patients with ovarian cancer, and this was felt to be mediated by more timely and earlier diagnoses [51]. Similarly, Medicaid expansion was shown to significantly decrease the 90-day mortality rate in patients with stage II and III rectal cancer [52]. Medicaid expansion has additionally been associated with a survival benefit in patients with hepatocellular carcinoma [34], gastric cancer [35], pancreatic adenocarcinoma [53,54], and intrahepatic cholangiocarcinoma [33]. Surprisingly, while there was an increase in the number of patients with Medicaid coverage in states that implemented Medicaid expansion after 2014, there was no corresponding impact on the stage at which genitourinary malignancies were diagnosed nor the survival rates of affected patients [55]. 

Medicaid expansion has been associated with survival benefits in patients across the age spectrum, including pediatric patients (age 0–14 years) and young adults (age 19–39 years). In a study of pediatric patients with cancer, there was a 1.5% (95% CI = 0.37 to 2.64) increase in the 2-year overall survival in expansion states relative to non-expansion states, particularly for those living in the lowest county income quartile [56]. In a National Cancer Database study of young adults (age 18–39 years) with cancer, the 2-year overall survival increased more in expansion states (90.39% pre-expansion to 91.85% post expansion) than in nonexpansion states (88.98% pre-expansion to 90.07% post expansion), resulting in a net increase of 0.55% (95% CI, 0.13 to 0.96) [57]. The expansion-associated survival benefits were most pronounced among patients of under-represented race and ethnicity and patients with high-risk diseases.

A recent study focusing on the impact of Medicaid expansion on breast cancer diagnoses and treatment in Southern states found that Medicaid expansion in the South led to earlier and more comprehensive treatment of breast cancer [58]. More specifically, among 21,974 patients in the North American Association of Central Cancer Registries (NAACCR) database from 2011 to 2018, patients in expansion states (Louisiana, Kentucky, and Arkansas) had decreased odds of being diagnosed with distant-stage breast cancer (odds ratio 0.93, *p* = 0.03), as well as higher odds of receiving treatment (odds ratio 2.27, *p* = 0.03) when compared to patients in non-expansion states (Tennessee, Alabama, Mississippi, Texas, and Oklahoma). Additionally, patients in expansion states were less likely to have been diagnosed with distant-stage disease when compared to patients in nonexpansion states.

Barnes et al. examined the impact of Medicaid expansion on cancer mortality rates and the importance of the stage at diagnosis [59]. This study utilized national databases spanning from 2001 to 2019. The findings revealed that, in Medicaid expansion states, there were greater reductions in both the rates of distant-stage cancer diagnoses and cancer death when compared to non-expansion states. Medicaid expansion resulted in 2591 fewer cases of cancer being diagnosed at an advanced stage, and 1616 fewer cancer-related deaths in expansion states between 2015 and 2019. Around 60% of the overall reduction in cancer mortality rates associated with Medicaid expansion was attributed to the decline in advanced-stage diagnoses. Furthermore, there were noticeable decreases in the breast, cervical, and liver cancer mortality rates that were specifically linked to the expansion of Medicaid.

**Figure 2:**
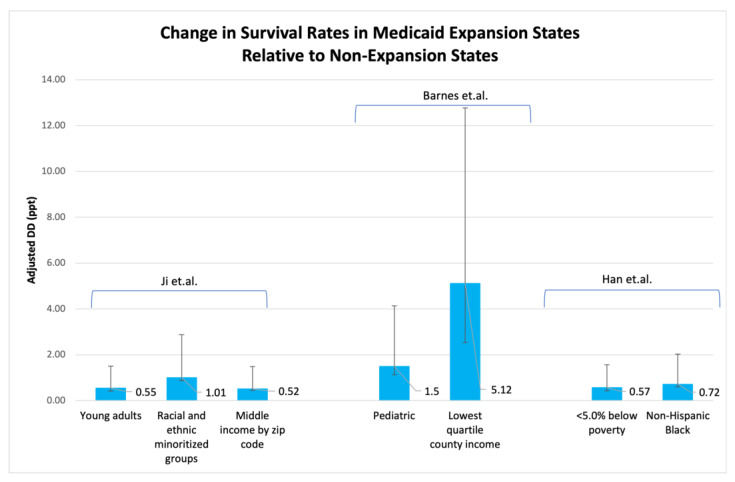
Significant improvements in the 2-year overall survival rates among specific sociodemographic groups of newly diagnosed cancer patients following Medicaid expansion. DD (for Ji et al. [57], Barnes et al. [56], and Han et al. [48]) and their 95% confidence intervals are presented for the following: young adults (age 18–39 years); racial and ethnic minoritized groups (including Hispanic, non-Hispanic Black, and other races and ethnicities); patients with middle income as determined by zip code (defined as 139–400% of federal poverty level); pediatric patients (age 0–14 years); patients with lowest quartile county income; patients at <5% below the poverty level; and Non-Hispanic Black patients. DD analyses were used to compare changes in survival in Medicaid expansion states relative to non-expansion states. DD: difference-in-differences; ppt: percentage point.

Lastly, according to a recent study [60], Medicaid expansion was linked to a greater reduction in the 2-year mortality rates among Black individuals with gastrointestinal cancers and who resided in states that implemented Medicaid expansion in comparison with their counterparts residing in states that did not expand Medicaid. Additionally, the authors of the study concluded that racial disparities in mortality either remained unchanged or worsened in nonexpansion states, while in expansion states they were largely mitigated across almost all cases. Furthermore, numerous other research efforts [48,56,57] have demonstrated the beneficial influence of Medicaid expansion on the survival rates among certain sociodemographic groups of newly diagnosed cancer patients, as depicted in Figure 2.

To summarize, the expansion of Medicaid has been associated with positive effects, but the impact noticeably varies across different types of cancer. Key areas of variation include the stage at which the cancer is diagnosed, the commencement of treatment, access to superior quality care, and survival rates, as highlighted in Table 1.

## 7. Limitations, Gaps, and Future Directions

While our understanding of the impact of Medicaid expansion on patients with cancer has significantly evolved, there remain limitations and gaps in the current research that necessitate further exploration. One of the most notable gaps, for example, is the lack of long-term data on health outcomes. Most studies focus on short-term data, often with follow-up periods lasting only a few years (on average between 2 to 5 years) after the implementation of the ACA, which limits our understanding of the enduring effects of Medicaid expansion on cancer outcomes, including survival rates, late-stage diagnoses, and quality of life. Longitudinal studies could help elucidate the continuing influence of Medicaid expansion on cancer care access, outcomes, and costs. Additionally, a better understanding of the financial impacts of Medicaid expansion on cancer care is needed. While the policy has undeniably increased access to care, comprehensive analyses of its financial implications, including potential cost savings and resource allocation, remain sparse.

Persistent disparities in terms of both access and outcomes also require further research. Medicaid expansion has largely had a positive impact, but substantial variations still exist among different population groups, including racial and ethnic minorities, rural residents, and individuals of lower socioeconomic status. Unraveling the factors behind these disparities and devising targeted interventions to counter them is a crucial area for future research. Geographic variability in Medicaid expansion policies further adds to these challenges, possibly limiting the applicability of findings from one state to another. Some states have fully embraced Medicaid expansion, while others have opted for partial expansions, or have decided against expansion altogether.

The influence of social determinants of health on cancer outcomes for Medicaid beneficiaries also warrants more investigation. A better understanding of these determinants might highlight how Medicaid expansion can mitigate their impact. Moreover, we need a better understanding of the specific mechanisms through which Medicaid expansion impacts cancer care. This includes exploring how increased health insurance access influences patient decision making, adherence to treatment plans, and overall quality of care. It is also necessary to account for potential confounding factors such as differences in treatment quality, patient demographics, and socioeconomic factors that could obscure the specific effects of Medicaid expansion.

Research on the impact of Medicaid expansion on patients with cancer has predominantly concentrated on access to care and treatment. Expanding the focus of future studies to include the policy’s impact on a broader spectrum of cancer-related services, such as palliative care, mental health services, and rehabilitation, is vital. Additionally, as novel therapies such as immunotherapies or targeted drug therapies advance, it is crucial that future research evaluates the extent to which Medicaid expansion enhances access to these cutting-edge treatments. It is particularly important to understand how, and if, Medicaid expansion allows those living in rural areas or those belonging to vulnerable populations to receive the benefits of these medical advancements.

Lastly, the increasing adoption of Medicaid-managed care models [61] by states presents a new frontier for research. Investigating these models’ impact on patients’ access to care, quality of care, and health outcomes could provide valuable insights into optimizing healthcare provisions under Medicaid. By bridging these gaps, hopefully a more comprehensive, nuanced, and ultimately more effective understanding of Medicaid expansion’s role in enhancing cancer care in the United States can be generated.

## 8. Conclusions

Despite the substantial body of research investigating the impacts of Medicaid expansion on cancer care, there are still inherent limitations and unresolved issues within the current literature. These gaps encompass the lack of comprehensive, long-term studies that engage with a multitude of variables, represent diverse populations, and encompass the full spectrum of cancer-related services. Addressing these gaps will not only deepen our understanding of the specific effects of Medicaid expansion on cancer care, but they will also provide invaluable insights that could guide future health policy decisions.

The expansion of Medicaid under the ACA has undeniably broadened health insurance coverage for millions of low-income Americans, including those contending with cancer. Yet, the precise impact of this policy on patients with cancer remains to be fully delineated. Our investigation underscores the need for continuing research in this area, allowing for the generation of an evidence-based approach to healthcare policy that is capable of optimizing outcomes for patients with cancer within the complex and evolving landscape of U.S. healthcare.

## Figures and Tables

**Table 1:** Exploring the Effect of Medicaid Expansion on Cancer Patient Outcomes: A Review of Key Studies.

Cancer Type/Patient Population	Key Findings	Implications of Medicaid Expansion
Breast cancer [30,39,45,50,58]	↑ Early-stage diagnoses ↑ 5-year survival ↑ Time to initiation of treatment for Black and Hispanic patients ↓ 2-year mortality gap for stage IV patients ↑ Comprehensive treatment in Southern states ↑ Rates of post-mastectomy breast reconstruction	Earlier detection, improved diagnosis and survival, faster treatment initiation, expanded treatment options
HNSCC Non-oropharyngeal HNSCC [31,40]	↑ Early-stage diagnoses ↑ Time to initiation of treatment for non-oropharyngeal HNSCC	Earlier detection, faster treatment initiation
Colorectal cancer [42]	↑ Early-stage diagnoses ↑ Perioperative outcomes	Earlier detection, improved surgical outcomes
Hepatocellular carcinoma [34]	↑ Early-stage diagnoses	Earlier detection
Gastric cancer [35]	↑ Early-stage diagnoses	Earlier detection
Kidney cancer [36]	↑ Early-stage diagnoses	Earlier detection
Pancreatic cancer [37,53,54]	↑ Early-stage diagnoses ↑ survival benefit	Earlier detection, improved survival rates
Intrahepatic cholangiocarcinoma [33]	↑ Surgical treatment	Increased treatment accessibility
Rectal cancer [52]	↓ 90-day mortality	Improved short-term survival
Gynecologic cancer (ovarian and endometrial) [32,49,51]	↑ Time to initiation of treatment ↑ Post-operative survival and 1-year survival due to earlier diagnoses for ovarian cancer	Faster treatment initiation, improved post-operative outcomes, improved early detection and survival
Genitourinary cancers [55]	No impact on diagnosis stage or survival rates	No significant impact
Melanoma [38,44]	↑ Early-stage diagnoses ↑ Receipt of therapies such as immunotherapy	Earlier detection, expanded treatment options
All cancer sites [47,48,59]	↑ In prognosis for specific cancer types ↓ Rates of distant stage cancer diagnoses and cancer death	Improved prognosis, improved diagnosis, and reduced mortality
Gastrointestinal cancers (Black individuals) [60]	↓ In 2-year mortality rates	Improved survival rates
Advanced/Metastatic cancers [41,43]	↓ Black–White racial disparities in treatment ↑ Receipt of timely systemic treatment	Decreased racial disparities, expanded treatment options
Cancer survivors [46]	↑ Access to smoking cessation assistance	Improved preventive care access
Pediatric and young adults [56,57]	Survival benefits	Improved survival rates

Abbreviations: HNSCC head and neck squamous cell carcinoma

## Data Availability

No new data were created or analyzed in this study. Data sharing is not applicable to this article.
